# Nitric Oxide Binding Geometry in Heme-Proteins: Relevance for Signal Transduction

**DOI:** 10.3390/antiox13060666

**Published:** 2024-05-29

**Authors:** Giovanna De Simone, Alessandra di Masi, Diego Sbardella, Paolo Ascenzi, Massimiliano Coletta

**Affiliations:** 1 Dipartimento di Scienze, Università degli Studi Roma Tre, 00146 Rome, Italy; giovanna.desimone@uniroma3.it (G.D.S.); alessandra.dimasi@uniroma3.it (A.d.M.); 2 Centro Linceo Interdisciplinare “Beniamino Segre”, Accademia dei Lincei, 00165 Rome, Italy; 3 IRCCS Fondazione Bietti, Rome, Italy; diego.sbardella@fondazionebietti.it; 4 Accademia Nazionale dei Lincei, 00165 Rome, Italy

**Keywords:** heme proteins, hemoglobin, myoglobin, soluble guanylate cyclase, cytochrome *c*, heme model compounds, nitric oxide, heme (protein) nitrosylation

## Abstract

Nitric oxide (NO) synthesis, signaling, and scavenging is associated to relevant physiological and pathological events. In all tissues and organs, NO levels and related functions are regulated at different levels, with heme proteins playing pivotal roles. Here, we focus on the structural changes related to the different binding modes of NO to heme-Fe(II), as well as the modulatory effects of this diatomic messenger on heme-protein functions. Specifically, the ability of heme proteins to bind NO at either the distal or proximal side of the heme and the transient interchanging of the binding site is reported. This sheds light on the regulation of O_2_ supply to tissues with high metabolic activity, such as the retina, where a precise regulation of blood flow is necessary to meet the demand of nutrients.

## 1. Introduction

The reaction of nitric oxide (NO) with heme proteins has been investigated since the pioneering studies of Pauling and coworkers [1], which predicted the paramagnetic properties of ferrous nitrosylated hemoglobin (Hb(II)-NO), paving the way for subsequent electron paramagnetic resonance (EPR) spectroscopic studies [2,3,4]. However, NO binding to heme proteins was not deeply investigated until the early 1980s, when this diatomic molecule was recognized as the long-time named endothelium-derived relaxation factor (EDRF) [5,6,7,8]. Since then, investigations on the role of NO and its binding mode to heme proteins have increasingly expanded, leading to a better understanding of its role as an important and multifunctional messenger [9,10,11].

NO has been demonstrated to play an important role in regulating blood pressure by initiating a cascade of events involving the heme proteins guanylate cyclase (sGC) and cyclic guanosine monophosphate (cGMP) [12,13,14]. NO binding to sGC induces an increase in cGMP formation, thereby amplifying the initial NO signal through a series of processes that further activate cGMP-dependent protein kinases, ion channels, and phosphodiesterases [15,16]. Therefore, the dysregulation of the NO-sGC-cGMP signaling pathway is associated with a variety of diseases, such as cancer and hypertension as well as cardiovascular and neurodegenerative diseases [17,18,19,20]. On the other hand, NO scavenging by various heme proteins, including hemoglobin (Hb) and myoglobin (Mb), can have contrasting effects by inducing vasoconstriction [21]. Therefore, NO may compete and/or cooperate with O_2_ binding to Hb and Mb, thereby magnifying its role as a messenger for the modulation of vasodilation and/or vasoconstriction, which relies on the intra-erythrocytic conditions [22].

The regulation of NO levels is crucial for maintaining adequate blood flow, oxygenation, and nutrient supply to poorly vascularized tissues. This is particularly relevant in tissues with high metabolic activity, such as the retina, where the precise regulation of blood flow is necessary to meet the demand of nutrients [23]. It is not surprising that alterations in NO levels and nitrosative stress have been found to play a crucial role in retinal and optic nerve pathologies, including diabetic retinopathy and glaucoma [19,24,25].

An additional effect that emphasizes the overall biological relevance of NO binding to heme proteins is the regulation of apoptosis. NO binding to cytochrome *c* (cyt *c*) has been demonstrated to affect the formation of the horse heart cyt *c* (hhcyt *c*)–cardiolipin (CL) complex (CL-hhcyt *c* complex). The CL-dependent release and translocation of heme proteins from mitochondria to the cytosol during the apoptotic cascade reflect the NO binding mode to the heme and the overall structural conformation adopted by hhcyt *c* [26,27,28].

Under most physiological conditions, globins react very rapidly with NO, either by binding it tightly or, in the presence of O_2_, irreversibly oxidizing it to nitrate. Consequently, examining all the NO reactions with globins is essential to understanding how these proteins regulate NO signaling and the role these processes play in detoxifying NO when it is inhaled or generated in excess due to inflammation. Globin genes have now been found in all kingdoms of life, and they often play a role in NO and O_2_ metabolism [29]. In this study, we focus on the interaction of NO with the reduced heme-Fe atom of some heme proteins. Specifically, we deeply analyze the interaction of NO with two prototypical globins (i.e., Hb and Mb), and with two prototypical heme proteins (i.e., sGC and cyt *c*). These four proteins are representative of the physiological and pathological significance of conformational transitions occurring in six-coordinated His-Fe(II)-NO and five-coordinated Fe(II)-NO complexes. To provide a comprehensive understanding of the structural–functional importance of NO binding geometry, we also mention the *Alcaligenes xylosoxidans* cytochrome *c*’ (*Ax*cyt *c*’) as it serves as an invaluable model system of sGC activation [30,31,32]. An understanding of NO interactions with heme proteins offers important insights into the molecular basis of NO signaling and opens avenues for therapeutic interventions targeting NO pathways in various diseases.

## 2. The Heme-Fe(II)-NO Binding Geometry

### 2.1. NO Binding to the Heme Distal Side: Hemoglobin and Myoglobin

Hb and Mb are considered molecular prototypes for the binding of gaseous ligands such as O_2_, CO, and NO to ferrous heme proteins. In particular, the binding mode of NO to the ferrous heme may, in principle, occur either through an Fe(II)-N-O geometry or with the formation of an Fe(II)-O-N complex, with Fe(II)-N-O being energetically much more favorable [33]. The Fe(II)-O-N geometry has never been observed in heme proteins but only in heme models after irradiation [34]; therefore, throughout this review, we refer only to the Fe(II)-N-O form.

In Hb(II)-NO, the bending angle appears to be optimal for the ligand to fit into the distal heme pocket without affecting the conformation of neighboring residues, including the distal heme His side chain [35]. This feature might account for the very high affinity of NO (as expressed by the equilibrium constant *J*) for Hb (*J*_R_ = 7.2 × 10^−13^ M at pH 7.0 and 20 °C) [36,37,38] and Mb (*J* = 7.1 × 10^−12^ M at pH 7.0 and 20 °C) [36,38] (see Table 1). Conversely, NO binding to six-coordinated heme proteins (e.g., HisF8-Fe(II)-HisE7 complexes) is limited from the cleavage of the distal Fe(II)-E7 bond. In mouse neuroglobin, the ligand- and concentration-independent rate for the cleavage of the distal Fe(II)-E7 bond preceding NO and O_2_ binding is 50 s^−1^ [39].

A further demonstration of the optimal fitting of NO into the heme distal pocket comes from the evidence that in Hb the NO association rate constant is essentially identical in the T- and the R-state [36] (see Table 1). This clearly indicates that quaternary-linked structural changes do not significantly affect the energetic barrier for the nitrosylation of the heme-Fe(II) atom. Therefore, the cooperativity of NO binding to Hb is fully exerted through a quaternary-linked variation of the NO dissociation rate constant [37,38] (see Table 1).

**Figure 1:**
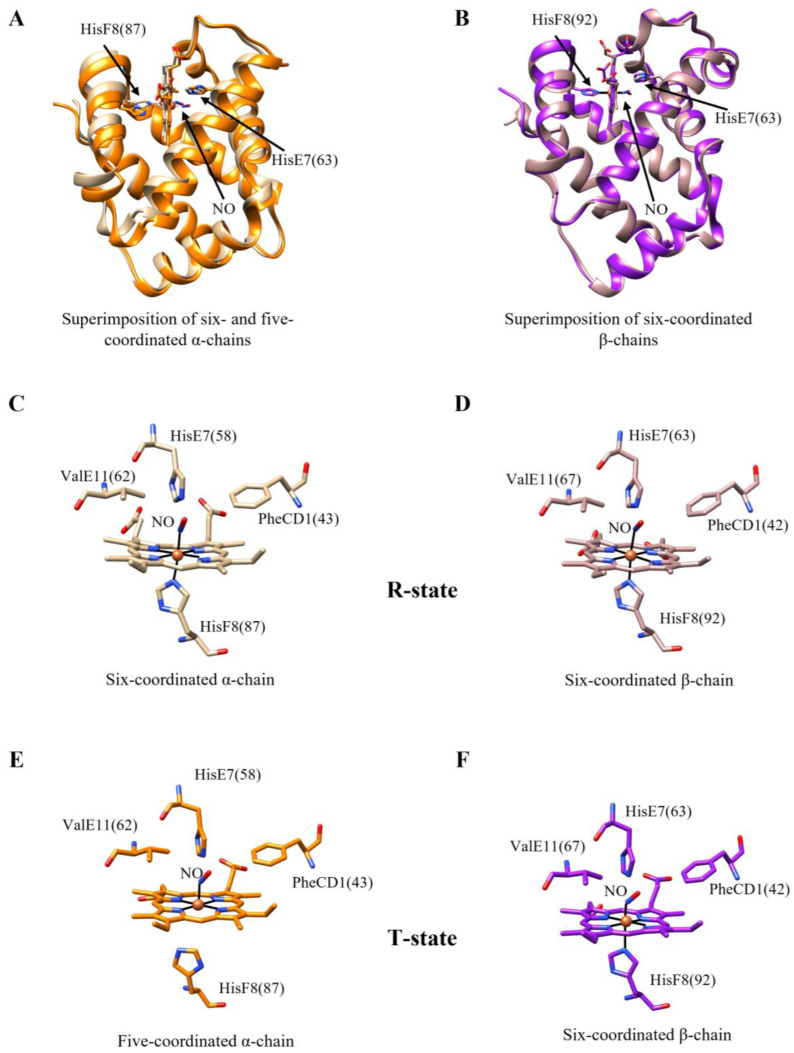
**IHP-induced allosteric transition of Hb(II)-NO.** (**A**) Superimposition of six- (in tan) and five-coordinated (in orange) α-chains (R- and T-state, respectively). (**B**) Superimposition of six-coordinated β-chains in the R- and T-state (in rosy brown and purple, respectively). (**C**,**D**) Three-dimensional structures of the heme-binding sites of ferrous nitrosylated α- and β-chains, respectively, of human Hb in the R-state. (**E**,**F**) Three-dimensional structures of the heme-binding sites of ferrous nitrosylated α- and β-chains, respectively, of human Hb in the T-state. PDB entries are 4N8T [60] and 1RPS [59]. Pictures have been drawn using the UCSF Chimera package [61].

A peculiar feature of Hb(II)-NO is the binding at multiple sites of inositol hexa-kis-phosphate (IHP), ATP, 2,3-di-phospho-glycerate (2,3-DPG), bezafibrate (BZF), and clofibrate (CFB), which induce the quaternary R_4_→T_4_ conformational transition. This leads to the cleavage (or severe weakening) of the proximal HisF8-Fe(II) bond (Figure 1, panels A and B) [40,41,42]. These effectors do not bind in the immediate surroundings of the heme but act as long-range intramolecular signal transducers. In particular, IHP, ATP, and 2,3-DPG bind to the α_1_α_2_- or β_1_β_2_-cavity of ligated Hb(II), while BZF and CFB recognize the CD corner and the inter-chain central cavity of the ligand-bound tetramer [43,44,45,46]. Moreover, the five-coordination of the heme-Fe(II)-NO adduct reflects the ligand occupancy of the β-hemes [47,48] and occurs upon lowering the pH, which induces the protonation of the N_ε_ atom of the proximal HisF8 residue [49]. Overall, the cleavage of the HisF8-Fe(II) bond is peculiar to Hb(II)-NO, as it is not observed in other Hb(II) ligated derivatives, such as Hb(II)-CO [50].

Binding of allosteric effectors to human Hb(II)-NO and the subsequent cleavage of the HisF8-Fe(II) bond (Figure 1, panels C–F) has been investigated by several techniques, including electronic absorption [51], circular dichroism [51], EPR [40,52,53], nuclear magnetic resonance (NMR) [54], resonance Raman (RR) [55,56], and infrared [57] spectroscopies. In particular, EPR spectra of Hb(II)-NO, which show a rhombic shape and a weak hyperfine pattern in the *g*_z_ region, display a dramatic change upon IHP binding. The appearance of three strong hyperfine splitting in the *g*_z_ region indicates the cleavage (or severe weakening) of the proximal HisF8-Fe(II) bond of the α-chains [40,52,53]. Further, electronic absorption spectra of five-coordinated Hb(II)-NO display a blue shift in the maximum wavelength in the Soret region and a drastic reduction in the extinction coefficient ε [41,51]. Additionally, the cleavage of the proximal HisF8-Fe(II) bond in Hb(II)-NO has been observed by RR spectroscopy, characterized by a decrease in the high-frequency region of a band at 1633 cm^−1^ (possibly attributed to the six-coordinated species) and the appearance of a band at 1644 cm^−1^ (likely reflecting the formation of a five-coordinated form) [55,56]. Overall, these observations indicate that the heme iron remains in the low-spin state after the cleavage of the proximal bond [51]. Therefore, this spectroscopic transition has been attributed to the quaternary R_4_→T_4_ transition in fully liganded Hb(II)-NO (Figure 1, panels A and B), reflecting the dramatic decrease in the donor interaction of NO with the distal imidazole ring [54].

The IHP-induced five-coordination of the metal center in fully liganded Hb(II)-NO appears to occur only in the α-subunits [58,59]. The structural basis for the different effects of NO binding to the α and β subunits (Figure 1, panels C–F) is related to the different intensity of the strain imposed by ligand binding in the T-state on the HisF8-Fe(II) proximal bond of the two subunits [49]. This strain reflects the strong repulsive *trans* effect generated by the unpaired electron of the axial NO ligand [49], leading to the rupture of the HisF8-Fe(II) proximal bond in the α subunits and, thus, to a larger movement of the heme-Fe(II) atom upon the interaction with NO. Thus, in the unliganded form of the α-chains, the Fe(II) atom is out of the heme on the proximal side by approximately 0.59 Å. NO binding displaces the Fe atom by about 0.84 Å toward the distal side, resulting in a final position of the Fe atom around 0.25 Å on the heme distal side of the heme. In contrast, in β chains, the heme-Fe(II) atom, initially positioned around 0.42 Å toward the proximal side, moves by only about 0.30 Å upon NO binding, remaining approximately 0.12 Å on the proximal side in the NO-ligated form [59]. This behavior clearly indicates: (*i*) a drastic difference in NO recognition between the two types of subunits, even without IHP, and (*ii*) the marked variation in the strength of the proximal HisF8-Fe(II) bond. In the fully liganded Hb(II)-NO, the p*K*_a_ value for the cleavage of the proximal HisF8-Fe(II) bond in α-nitrosylated hemes is about 5.8, while in β-nitrosylated hemes, the p*K*_a_ value is 3.8.

This difference suggests an 11.4 kJ/mol difference in the proximal bond energy between the two chains [48].

From the functional standpoint, the subunit heterogeneity in Hb(II)-NO is reflected in the diatomic-ligand dissociation rate constants. This is already evident in the R-state, as observed in the α_1_β_1_ (or α_2_β_2_) dimers complexed with haptoglobin (Hp) (see Table 1), wherein a two-fold difference occurs between the α and β subunits [62]. This difference was previously obscured in measurements because it overlapped with the quaternary-linked conformational change [37,38]. The addition of IHP, accompanied by the R_4_→T_4_ quaternary transition and the consequent five-coordination of the α-NO subunits, resulted in a much more evident functional heterogeneity [38] of the α and β subunits; the rate enhancement of NO dissociation is much more pronounced for the β-subunits than for the α-chains [63]. Therefore, since no evidence of chain heterogeneity for NO binding in the T-state has been reported previously, these data underline that α-chains have a higher affinity for NO in the T conformation. Thus, α-chains become preferentially occupied at low NO concentrations because the cleavage of the proximal HisF8-Fe(II) bond leads to a slower NO dissociation rate constant. This feature is confirmed by equilibrium measurements of NO binding to Hb(II) both in the absence and in the presence of IHP, wherein after an initial similar occupation by NO of α- and β-hemes (due to the similar association rate constant), a slow re-equilibration occurs in favor of the five-coordinated α-subunits [64]. Therefore, data from Refs. [63,64] clearly suggest that the different NO dissociation rate constants of ferrous nitrosylated α- and β-hemes are mostly due to the proximal strain rather than to the different amino acid residues lining the heme pocket of Hb subunits.

This functional effect is also observed in the interaction of human Hb(II)-NO with efaproxiral, a NO-releasing pro-drug, which has been shown to reduce O_2_ affinity, facilitating O_2_ release to organs and tissues [65]. Interestingly, this effect is coupled with NO binding to the proximal side of the heme pocket of Hb(II) α-subunits in the T-state; notably, only α-subunits are nitrosylated in the bis-NO-ligated tetramer [65]. Although accurate thermodynamic data are not available yet, spectroscopic data suggest that NO binding to tetrameric Hb(II) at neutral pH shows a stabilization of the T-liganded structure up to relatively high ligand saturation degrees (even in the absence of allosteric effectors); therefore, the addition of IHP brings about a predominance of the T-liganded form, even for fully saturated Hb(II)-NO [65].

Similarly, Mb(II)-NO displays spectroscopic changes similar to those of Hb((II)-NO, but only after lowering the pH down to about 4.0 [66,67,68] or replacing the proximal HisF8 residue with a non-coordinating residue, such as Gly in the site-directed mutant His93Gly [49]. A full spectroscopic characterization of the five-coordinated Mb(II)-NO species highlights a pure three-line pattern in the high-frequency region of the EPR spectrum [49,66,67]. In parallel, the maximum wavelength in the Soret region displays a blue shift (<414 nm) compared with the peak absorption at 421 nm of the six-coordinated Mb(II)-NO [49,66,68]. Interestingly, the relaxation time of the reversible six-to-five transition was estimated to be approximately 0.3 ms [66].

Similarly to Hb and Mb, the ferrous nitrosylated derivative of the neuronal isoform of rat nitric oxide synthase (NOS) undergoes the reversible six-to-five transition of the heme-Fe(II)-NO adduct under physiological conditions. Thus, the EPR spectrum of the substrate-free NOS is typical of a five-coordinated heme-Fe(II)-NO complex, whereas binding of the substrate L-Arg and of its derivatives Nω-monomethyl-L-Arg and Nω-hydroxy-L-Arg induces the appearance of a hexa-coordinated metal center. The perturbation in the EPR spectrum indicates that the heme-Fe(II)-NO coordination structure is altered upon binding of L-Arg and its derivatives to the heme distal site very close to NO [69].

**Table 1:** Values of *j*_on_ and *j*_off_ for the (de)nitrosylation of Hb(II)(-NO), Hp1-1:Hb(II) (-NO), Hp2-2:Hb(II) (-NO), α(II)(-NO), β(II)(-NO), and Mb(II)(-NO) species.

Heme Protein	*j*_on_ (M^−1^ s^−1^)	*j*_off_ (s^−1^)	*j*_off1_ (s^−1^)	*j*_off2_ (s^−1^)
Hb(II)(-NO) + IHP (T-state)	2.5 × 10^7 a^		3.1 × 10^–3 b^ 1.1 × 10^–3 c^	9.0 × 10^–5 b^ 4.2 × 10^–4 c^
Hb(II)(-NO) (R-state)	2.5 × 10^7 a^	1.8 × 10^–5 b^		
Hp1-1:Hb(II)(-NO)	1.1 × 10^7 d^		6.4 × 10^–5 e^	3.6 × 10^–5 e^
Hp1-1:Hb(II)(-NO) + IHP			6.3 × 10^−5 e^	3.1 × 10^−5 e^
Hp2-2:Hb(II)(-NO)	9.3 × 10^6 f^		5.8 × 10^–5 e^	3.1 × 10^–5 e^
Hp2-2:Hb(II)(-NO) + IHP			6.3 × 10^−5 e^	3.5 × 10^−5 e^
α(II)(-NO) chains ^a^	2.4 × 10^7^	4.6 × 10^−5^		
β(II)(-NO) chains ^a^	2.4 × 10^7^	2.2 × 10^−5^		
Mb(II)(-NO) ^a^	1.7 × 10^7^	1.2 × 10^−4^		

^a^ pH 7.0 and 20.0 °C [37]. ^b^ pH 7.0 and 20.0 °C [38]. ^c^ pH 7.4 and 20.0 °C [63]. The slower rate refers specifically to five-coordinated α-chains of tetrameric Hb(II)NO. ^d^ pH 7.5 and 20.0 °C [70]. ^e^ pH 7.0 and 20.0 °C [62]. ^f^ pH 7.6 and 20.0 °C [70].

### 2.2. NO Binding to the Heme Proximal Side: Axcyt c′

Cyt *c*’ are a unique family of class IIa cytochromes located in the periplasm of specific denitrifying, nitrogen-fixing, photosynthetic, methanotrophic, and sulfur-oxidizing bacteria [71,72,73]. Traditionally thought to be involved in electron transfer, it has been suggested that cyt *c*’ in denitrifying bacteria may bind nitric oxide (NO), helping to alleviate nitrosative stress [74,75,76].

Cyt *c*’ forms homodimers, with each subunit consisting of an antiparallel four-helix bundle of about 130 residues containing a *c*-type heme [77]. A conserved Cys-Xxx-Xxx-Cys-His motif encompasses the proximal His120 residue as well as the Cys116 and Cys119 side chains, which form thioether bonds with the heme. The sixth coordination site on the heme distal face is accessed by the side chain of a hydrophobic residue (Leu16), making the distal pocket sterically crowded, hydrophobic, and less accessible to water solvent. In contrast, the proximal pocket is less crowded, positively polarized, and more solvent-accessible [32].

**Figure 2:**
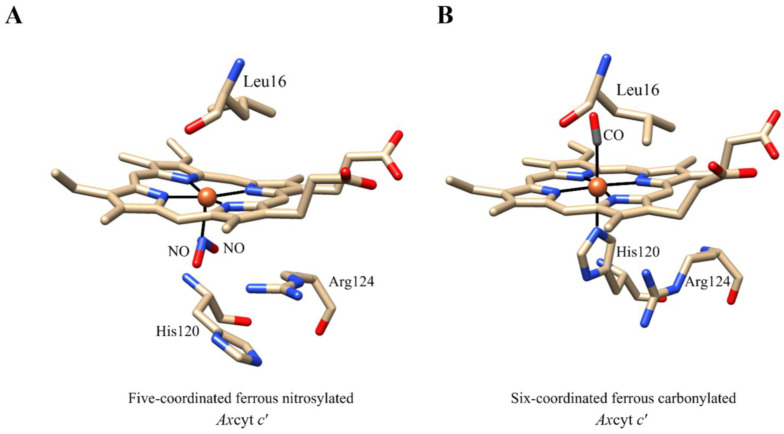
**Three-dimensional structure of the heme-binding site** of (**A**) ferrous nitrosylated and (**B**) ferrous carbonylated *Ax*cyt *c*’. PDB entries are 1E86 and 1E85 [31]. Pictures have been drawn using the UCSF Chimera package [61].

Under physiological conditions, cyt *c*’ appears to act with the heme iron in its ferrous state, which binds NO and CO [73]. Specifically, EPR spectra of NO-bound cytochromes *c*’ display features characteristic of the five-coordinated species with the cleaved His120-Fe(II) proximal bond [78]. NO binds to the proximal side of the *Ax*cyt *c*’ metal center, forming a five-coordinated Fe(II)-NO complex (Figure 2, panel A). On the other hand, CO binds to the heme distal site, resulting in the six-coordinated His-Fe(II)-CO species (Figure 2, panel B) [31,79].

Nitrosylated *Ax*cyt *c*’ exhibits two equally populated conformers with an average Fe-N-O angle of 128°. The structures of ferrous ligand-free and nitrosylated derivatives of *Ax*cyt *c*’ match very well. The proximal axial ligand of the heme iron is His120 in the ligand-free form and NO in the nitrosylated derivative. Upon NO binding, Arg124 undergoes large conformational changes, stacking against the heme plane and forming a hydrogen bond with conformer 2 of NO. In both ferrous ligand-free and nitrosylated derivatives, the heme-Fe atom is out of the heme plane by about 0.3 Å toward the proximal axial ligand (i.e., His120 in the ligand-free form and NO in the nitrosylated form) [31].

**Figure 3:**
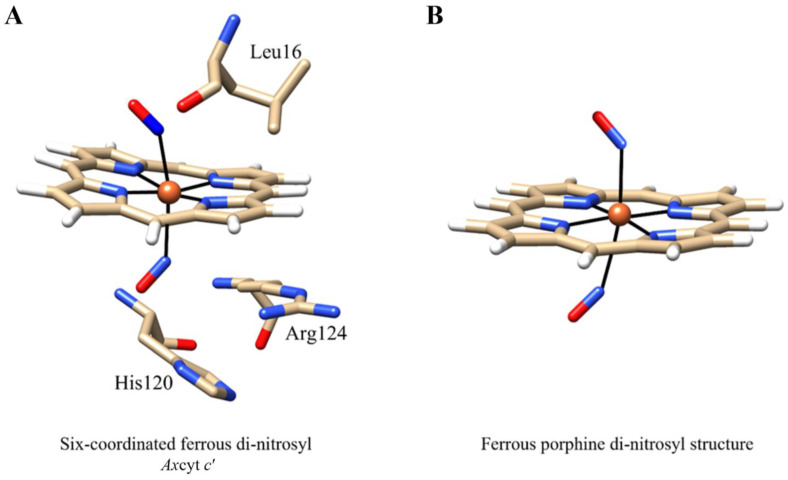
**Ferrous di-nitrosylated hemes.** (**A**) Three-dimensional model of the heme-binding site of the di-nitrosyl derivative of ferrous *Ax*cyt *c*’ (modified from ref. [32]). (**B**) Computed ferrous porphirine di-nitrosyl structure (modified from ref. [93]). The figures have been drawn using the UCSF Chimera package [61].

Nitrosylation of ferrous *Ax*cyt *c*’ is a biphasic process, with both phases being dependent on the NO concentration. Thus, after the initial formation of a six-coordinated species, likely referable to the His-Fe(II)-NO form, a five-coordinated species emerges [80]. At pH 8.9, the two bimolecular rate constants are *j*_1_’ = 4.4 × 10^4^ M^−1^ s^−1^ for the formation of the initial six-coordinated species and *j*_2_’ = 8.1 × 10^3^ M^−1^ s^−1^ for the transition from the six- to the five-coordinated form [81]; the bimolecular character of both reactions suggests a multi-step mechanism, with the final five-coordinated species resulting from a competitive process where NO prevails due to its higher reactivity and affinity (see below). Of note, the value of the rate constant depicting the first process is 100–1000-fold slower than that reported for other heme proteins, such as Mb [36], CL-hhcyt *c* [82], and zebrafish nitrobindin [83]. Similarly, CO binding to *Ax*cyt *c*’ has been reported to be very slow (*j* = 9.2 × 10^1^ M^−1^ s^−1^) [84], indicating a severe steric barrier for NO and CO binding from the distal side of the heme pocket [31]. Furthermore, the RR spectroscopic features of the intermediate six-coordinated species show a frequency value of ν(Fe-NO) (=579 cm^−1^) much higher than that observed for other six-coordinated Fe(II)-NO heme-proteins, where values ranging between 536 cm^−1^ and 554 cm^−1^ have been observed [81]. This indicates that the six-coordinated species has a grossly distorted Fe(II)-NO bond, with an Fe-N-O angle well below the typical value of ~140° exhibited by unhindered Fe(II)-NO hemes [85], likely reflecting the presence of Leu16 in the distal side of the heme pocket. In fact, Leu16 must be grossly displaced to accommodate NO into the crowded distal site [86], as also observed for the X-ray structure of the CO-bound derivative of *Ax*cyt *c*’ [31]. Therefore, the nitrosylation of ferrous *Ax*cyt *c*’ on the distal side leads to the weakening of the His120-Fe bond, facilitating a second NO to displace the proximal His120 residue [32,87] (Figure 3, panel A).

*Ax*cyt *c*’ nitrosylation has been hypothesized to occur through a six-coordinated NO-Fe(II)-NO intermediate, leading to the final five-coordinated proximal Fe(II)-NO adduct [88]. The transient NO-Fe(II)-NO complex has never been observed, suggesting that NO binding to the proximal side and NO dissociation from the distal side are much faster processes than the cleavage of the proximal N_ε_His120-Fe(II) bond, which would then represent the rate-limiting step of the whole process [86].

The Arg124 residue at the proximal side of the heme pocket plays a critical role in NO recognition. In the ligand-free reduced state of *Ax*cyt *c*’, the side chain of Arg124 lies parallel to the proximal His120. However, in the five-coordinated nitrosylated species, Arg124 is hydrogen bonded to the proximal NO, and the imidazole ring of His120 is stacked against the heme plane [31]. Hybrid quantum/classical simulations indeed suggest that the simultaneous presence of His120 and Arg124 imposes a steric constraint within the heme pocket, weakening the His120-Fe(II) bond that appears to be already almost cleaved upon NO binding to the heme distal side (Figure 3, panel A) [32]. As the structure relaxes toward an energy minimum, the His120-Fe(II) bond breaks down, allowing NO to bind to the proximal side. A competition exists between NO and His120 for the axial proximal coordination that favors NO, which is responsible for the slow bimolecular second step (see above). The proximally bound Fe(II)-NO complex is stabilized by (*i*) the direct interaction of Arg124 with NO, which slows down the NO dissociation rate constant, and (*ii*) the strain induced by Leu16 on the distally bound NO, weakening its interaction with the heme-Fe(II) atom [89]. Therefore, the proximally bound NO is at an energy minimum [32].

Although not observed in heme proteins, the NO-Fe(II)-NO adduct has been envisaged in ferrous porphyrins of the type Fe(II)(TPP) (TPP = *meso*-tetraphenyl-porphyrin). These heme models bind reversibly to NO, leading to the formation of the paramagnetic mono-nitrosyl complex Fe(II)(TPP)(NO) [90]. However, under an excess of NO (i.e., 400 Torr of NO) and low temperature (i.e., 120 K), the formation of the dinitrosyl complex (Fe(II)(TPP)(NO)_2_ has been suggested by the disappearance of the EPR spectrum [91]. The reversible formation of Fe(II)(TPP)(NO)_2_ has been also reported at low-temperature solutions by NMR, IR, and UV-Vis spectroscopy [92].

NMR data indicate that the Fe(II)(TPP)(NO)_2_ complex is diamagnetic [92], and IR spectra point to a centrosymmetric *trans*-di-nitrosyl configuration [93]. Further spectroscopic and density functional theory (DFT) studies indicate that the preferred geometry of the Fe(II)(TPP)(NO)_2_ complex is a *trans–syn* conformation. In fact, the two NO molecules on the opposite side of the heme are bending in the same direction with a very small dihedral angle between the planes defined by the two Fe-N-O angles, 2.2° and 2.1°, respectively [93], a conclusion confirmed also by molecular orbital calculations [94] (Figure 3, panel B).

## 3. Relevance of the NO Binding Geometry in Physiological Processes

### 3.1. The Activation of the Soluble Guanylate Cyclase

**Figure 4:**
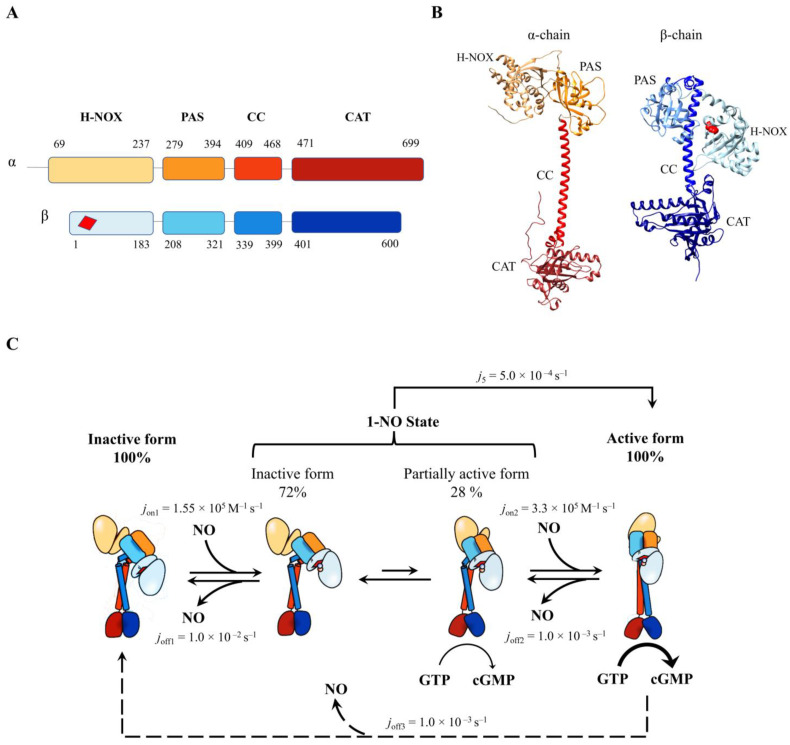
**Structural organization and NO-dependent catalytic mechanism of sGC**. (**A**) Schematic representation of the sGC heterodimer domains. H-NOX, heme nitric oxide/oxygen; PAS, Per/Arnt/Sim (PAS)-like domain; CC, coiled-coil domain; CAT, catalytic cyclase domain. The heme is represented by the red quadrilateral in the β H-NOX domain. (**B**) Three-dimensional model of the α1 (Uniprot entry: Q9ERL9) and β1 subunits (Uniprot entry: O54865) of mouse sGC. The His105 residue is represented by the red spheres. (**C**) sGC activation pathway. Upon the addition of NO, an equilibrium exists between the inactive form (72%) and the partially active form (28%). Further addition of NO or stimulator compounds (e.g., YC-1, 3-(5′-hydroxymethyl-2′-furyl)-1-benzylindazole) shifts the equilibrium toward the 100% active sGC form (modified from ref. [99]). The dashed line indicates the dissociation of NO from the active form of sGC, with the consequent inactivation of the enzyme. In the lower and upper panels, the sGC domains are represented by the same colors.

The cytosolic protein sGC is a ~150 kDa heterodimer consisting of one α- and one β-subunit (660 and 607 residues, respectively), with dimerization being essential for the conversion of GTP to cGMP [95]. Each subunit contains four major domains: (*i*) the heme NO/O_2_- (H-NOX)-like *N*-terminal sensor domain, which in the β-subunit houses the heme binding site; (*ii*) the Per/Arnt/Sim (PAS)-like domain; (*iii*) the coiled-coil (CC) domain; and (*iv*) the *C*-terminal catalytic (CAT) domain, which facilitates the conversion of GTP to cGMP (Figure 4, panels A and B) [96,97].

In the heme-unliganded species, also known as the inactive form, sGC adopts a bent coiled-coil structure arranged in a pseudo-two-fold symmetric manner, with the two PAS domains at the center and the two H-NOX domains positioned at opposite sides (Figure 4, panel B) [98,99]. The heme-Fe atom is coordinated to the His105 residue of the F-helix of the β-subunit. However, the H-NOX domain of the α-subunit does not bind heme due to the absence of the coordinating His and the occupation of the putative heme pocket by the *N*-terminal extension [98,99]. Together, the two PAS domains and the two H-NOX domains constitute the *N*-terminal sensor module of sGC. The CC domains dimerize to form the transducer module, which adopts a bent conformation in the inactive state and extensively contacts the CAT domains. The CAT domains of the two subunits are organized in a pseudo-symmetric manner, but in the inactive state, the angle between these domains results in a steric clash between the substrate and protein residues, impairing substrate binding (Figure 4, panel B) [98,99].

Since the activation of sGC occurs upon NO binding to the ferrous heme, the unliganded reduced form of sGC is maintained by a positive redox potential (i.e., +187 mV for the full-length bovine sGC) [100]. Interestingly, H_2_S has been proposed to play a role in rescuing sGC(II) by reducing the heme back to the ferrous oxidation state in the case of undesired oxidation [101]. NO binding to sGC(II) induces a shift in the absorption spectrum peak wavelength from 431 nm to 399 nm, being accompanied by the appearance of an EPR spectrum characterized by a three-line splitting [102,103]. Taken together, these findings suggest that sGC(II)-NO, similarly to what is observed in Hb upon the addition of IHP [44], possesses a five-coordinated heme equilibrium state. This is attributed to the cleavage of the proximal His105-Fe(II) bond due to the strain on the proximal side, which can be detected by RR spectroscopy even in the unliganded sGC(II) form [104]. This possibly reflects the proximity of the F-helix of the β-subunit H-NOX domain and the CC domain of the same subunit, which interact closely through the PAS domain [98].

Distortion of the proximal bond is somehow amplified and energetically favored by allosteric stimulators that cooperate with NO to activate the conversion of GTP to cGMP. Among these, benzylindazole (YC-1), the first stimulator discovered, can enhance the activity of sGC at low concentrations of NO with positive co-cooperativity [99,105]. YC-1 binds to a cleft between the β-H-NOX domain containing the heme and the CC module of the β-subunit, facilitating the interaction between the two domains and promoting the stabilization of the sGC active conformation [105,106]. In detail, the phenyl ring of YC-1 contacts Tyr112, thereby favoring interactions that lower the energy barrier for the cleavage of the proximal His112-Fe(II) bond. The most relevant interactions are established between (*i*) Asp106 of the β-H-NOX domain and the Arg305 and Lys307 residues of the PAS domain; (*ii*) Lys 307 of the PAS domain and Asp411 of the CC module; and (*iii*) Arg258 of the PAS domain with Asp102 of β-H-NOX [106]. In contrast to stimulators, sGC activators (e.g., BAY 58-2667, HMR1766, and cinaciguat) bind to the heme pocket of sGCs, replacing the heme. Notably, they do not promote enzyme turnover in synergy with NO but trigger cGMP synthesis in oxidized or heme-deficient sGC [105].

As reported for the α-subunits of Hb in the presence of IHP [44], the heme-Fe atom of sGC(II)-NO is pulled toward the distal side, intensifying the strain on the proximal side. This leads to the cleavage of the His105-Fe(II) bond. The F-helix is now free to rotate, causing a disruption of the hydrogen-bond network with residues of the CC domain. This allows the CC domain to undergo a transition from the bent conformation (in the unliganded inactive form) to a straight conformation (in the NO-bound active form). Then, the CC domain induces a 17° rotation of the α-subunit of the CAT domain with respect to the β-subunit [98], permitting GTP binding at the central pocket between the two catalytic domains and the subsequent cyclization to cGMP [99,107].

The molecular details of the NO-induced sGC activation are quite complex and still unclear. NO binding is characterized by two bimolecular phases: (*i*) a very fast one, corresponding to the formation of the six-coordinated species His-Fe(II)-NO (*j*’_1_ > 1.4 × 10^8^ M^−1^ s^−1^), and (*ii*) a slow one attributed to the cleavage of the proximal His105-Fe(II) bond with the consequent formation of a five-coordinated Fe(II)-NO species (*j*’_2_ = 2.4 × 10^5^ M^−1^ s^−1^) [108]. These data clearly indicate that the activation of sGC(II) by NO occurs via a two-step process, with both steps being dependent on the NO concentration (Figure 4, panel C). The existence of a six-coordinated intermediate is supported by EPR spectroscopy; thus, when NO binds to sGC(II) at −24 °C in glycerol and ethylene glycol, the transient formation of a six-coordinated species is observed, followed by the appearance of a five-coordinated form [106]. The bimolecular nature of the slow process suggests that the cleavage of the His105-Fe(II) bond (see below) involves an additional NO-binding step. These NO-dependent species are related to the sGC activity. Indeed, in the unliganded sGC(II) form, no activity is observed, while upon addition of ~one equivalent of NO (i.e., a condition in which most of the sGC(II)-NO is in the six-coordinated form), approximately 28% activity is observed. Full activity is achieved with an excess of NO, when all the sGC(II)-NO exists in the five-coordinated form [109]. Notably, the biphasic NO binding process occurs only in the full sGC, as the isolated H-NOX domain shows only a single NO binding phase that leads to a five-coordinated Fe(II)-NO form, the rate constant being slightly faster than that of the first step in the heterodimeric sGC [108]. Thus, the intermediate six-coordinated species is likely stabilized by the interaction of other domains with the H-NOX domain, where heme is located [96,97].

Currently, three models describe the two-step activation process: (*i*) the second-binding-site model, (*ii*) the NO flip model, and (*iii*) the kinetic model.

The second-binding-site model, the simplest one of the three, suggests the presence of a second low-affinity binding site for NO in the sensor domain of sGC in addition to the heme. One possibility is the involvement of a surface Cys residue in sGC, which could react with NO to form a transient RS-NO^●−^ radical anion [110]. However, specific Cys residues responsible for NO binding have not yet been identified, and it remains unclear how the nitrosylation of surface cysteine(s) could switch sGC to the fully activated state. Nevertheless, Cys489 and Cys571 residues (located in the β-subunit) might participate in sGC action(s), providing some support to the second-binding-site model. This introduces the concept that sGC activity can be mediated by thiol/disulfide switches [111].

The NO flip model suggests that NO binds to the proximal side of the heme in the fully activated form of sGC. This model is inspired by studies on H-NOX domains, which, upon addition of excess NO, were crystallized in the five-coordinated Fe(II)-NO form with NO in the proximal heme pocket [112], as observed for cytochrome *c*’ [30]. This model received support from a rapid freeze–quench EPR spectroscopic study, indicating that NO can indeed coordinate to the proximal side of the heme in full-length sGC [113]. This seems to confirm the model proposed by Russwurm and Koesling [114] indicating that the distal five-coordinated Fe(II)-NO complex displays low sGC activity at low NO levels. Conversely, when the NO concentration increases, the initial His-Fe(II)-NO intermediate seems to convert to a fully active enzyme displaying NO as the proximal coordination bond of the heme-Fe(II) atom. However, it is important to note that the EPR experiment mentioned does not directly distinguish between NO bound to the proximal and distal sides of the heme. Therefore, it remains unclear whether the two observed forms correspond to NO molecules bound to opposite sides of the heme or if they reflect two different geometries of the five-coordinated heme with the distally bound NO. The investigation by very fast time-resolved absorption spectroscopy on the motion of the proximal His105 residue in sGC when NO is present suggested that the proximally bound five-coordinated NO complex is not relevant under physiological conditions and may be an artifact of the experimental conditions used [115]. Furthermore, double electron–electron resonance (DEER-EPR) spectroscopic experiments, which measure the distance between the heme-bound NO in the five-coordinated complex and a spin label attached to the Cys17 residue, indicated that the predominant species in solution is the five-coordinated Fe(II)-NO complex with NO bound at the distal pocket [116].

In conclusion, it seems that the solid-state structures with NO in the proximal pocket [110] might originate from favorable crystallization conditions. Moreover, the formation of the proximal five-coordinated Fe(II)-NO complex in vitro may occur at high, non-physiological concentrations of NO, while the distal complex is favored at physiological NO concentrations [116].

The last model postulated for the full activation of sGC is the so-called “kinetic model”, which represents a compromise between the two previously described models [117]. After the initial rapid binding of NO, which forms the intermediate six-coordinated His105-Fe(II)-NO species with low cGMP formation activity, the *trans* NO pulling effect on the heme-Fe atom weakens the proximal His-Fe bond. This leads to an equilibrium between a distorted proximal-bond six-coordinated species and a five-coordinated distal heme-Fe(II)-NO complex. At low NO concentration ([NO]_free_ ≤ 5–10 μM), the amount of the five-coordinated species is relatively low, keeping cGMP activity at an intermediate level. The increase in free NO concentration ([NO]_free_ > 10 μM) leads to a NO-dependent increase in cGMP activity. Then, NO competes effectively with the re-binding of His105 to the proximal side, driving the second NO-dependent kinetic process, which results in the formation of a transient NO-Fe(II)-NO complex. Although the binding of NO to the proximal side is fast enough to kinetically impair the re-formation of the inactive His105-Fe(II)-NO form, maintaining high cyclase activity, it does not establish a stable equilibrium, likely due to a rapid dissociation rate constant. Noteworthy, similar NO-dependent kinetics were observed for the conversion of the six-coordinated Fe(II)-NO species to the corresponding five-coordinated derivative in Cyt P460 [118]. Therefore, according to the “kinetic model”, the second NO acts as a “catalyst”, promoting the formation of the five-coordinated distally bound Fe(II)-NO complex and thereby enhancing cGMP activity through an indirect dynamic mechanism, which indeed envisages a fine-tuning by NO levels. In fact, a decrease in free NO concentration results in the re-formation of the six-coordinated His105-Fe(II)-NO species and the deactivation of sGC cyclase activity [115].

Accordingly, the enzymatic activity of sGC is controlled by NO levels in a very precise manner, thanks to an unstable active conformation that is dynamically switched “on” and “off” rather than being in a stable equilibrium [119].

Finally, the distorted His105-Fe(II) bond that undergoes cleavage upon NO binding, triggering the “two-step” mechanism with kinetic stabilization of the five-coordinated Fe(II)-NO species, is likely also responsible for the positive selectivity of sGC(II) for NO with respect to O_2_ and CO. These two ligands show a low affinity for the His-bound five-coordinated heme, while the His105-Fe(II) metal center displays a high affinity for NO [63,64,120,121]. However, CO induces a small but significant sGC-catalyzed cyclic activity due to the ligand-dependent cleavage of the His105-Fe(II) bond [122]. This opens new fields of investigation given that CO is a molecule with roles in physiological signaling cascades [123].

### 3.2. NO Binding to Cytochrome c and to Its Complex with Cardiolipin

Eukaryotic cyt *c* are globular heme proteins placed within the inner and outer mitochondrial membranes [72]. Hhcyt *c* is a monomeric heme protein made up of 104 amino acids structured in five α-helices connected by Ω-loops [124]. The six-coordinated heme of hhcyt *c* lies inside a cavity, being covalently bound to Cys14 and Cys17 residues by thioether bonds. Although His18 and Met80 axially coordinate the metal center, hhcyt *c* binds NO, but not CO and O_2_ [125]. NO-bound hhcyt *c* displays EPR spectra typical of a His18-Fe(II)-NO species [126], indicating that NO can displace Met80 as the sixth axial ligand. Of note, the affinity of NO for the six-coordinated native hhcyt *c* is lower compared with that for carboxymethylated hhcyt *c* (i.e., *J* decreases from 8.2 × 10^−6^ M for native hhcyt *c* to ≤5 × 10^−8^ M for carboxymethylated hhcyt *c* at pH 7.0 and 10.0 °C) [127]. Remarkably, the heme of carboxymethylated hhcyt *c* is five-coordinated because of the Met80 displacement by an alkylating reagent [128,129]. The reaction of NO with the heme-Fe atom in native hhcyt *c* is accompanied by the formation of an *S*-NO group, which might eventually facilitate the binding of NO to the metal center of hhcyt *c*. The *S*-nitrosation might involve Cys14, which connects covalently the heme with the protein moiety through a thio-ether bond [130].

Cyt *c* is involved in electron transfer from the *bc*_1_ complex to the terminal acceptor cytochrome *c* oxidase, and in triggering the apoptosis program [131,132]. In non-apoptotic cells, cleavage of the Met80-Fe bond induces the translocation of cyt *c* from the intermembrane space to both the nucleus and cytoplasm [133]. In contrast, the very early phases of apoptosis induction are linked to the interaction of cyt *c* with CL before it is released from the mitochondrion [134,135,136]. CL, which constitutes about 20% of the total lipids of the mitochondrial membrane, is synthesized in the mitochondrion and interacts with cyt *c* through its two acyl chains, inducing significant conformational change(s) at both the proximal and distal sides of the heme. This interaction results in the loss of the electron-transfer properties of hhcyt *c* due to the cleavage of the Met80-Fe bond, converting the five-coordinated reduced form to one that binds CO and NO with high affinity [137,138]. Furthermore, the CL-hhcyt *c* complex displays peroxidase activity [139,140], which also induces the permeabilization of the mitochondrial membrane through the peroxidation of CL [141]. Therefore, the CL-hhcyt *c* complex is an important modulator of apoptosis, and NO and/or CO binding may play a significant anti-apoptotic role by inhibiting the peroxidase activity of the CL-hhcyt *c* complex [140,142,143,144].

**Scheme 1:**
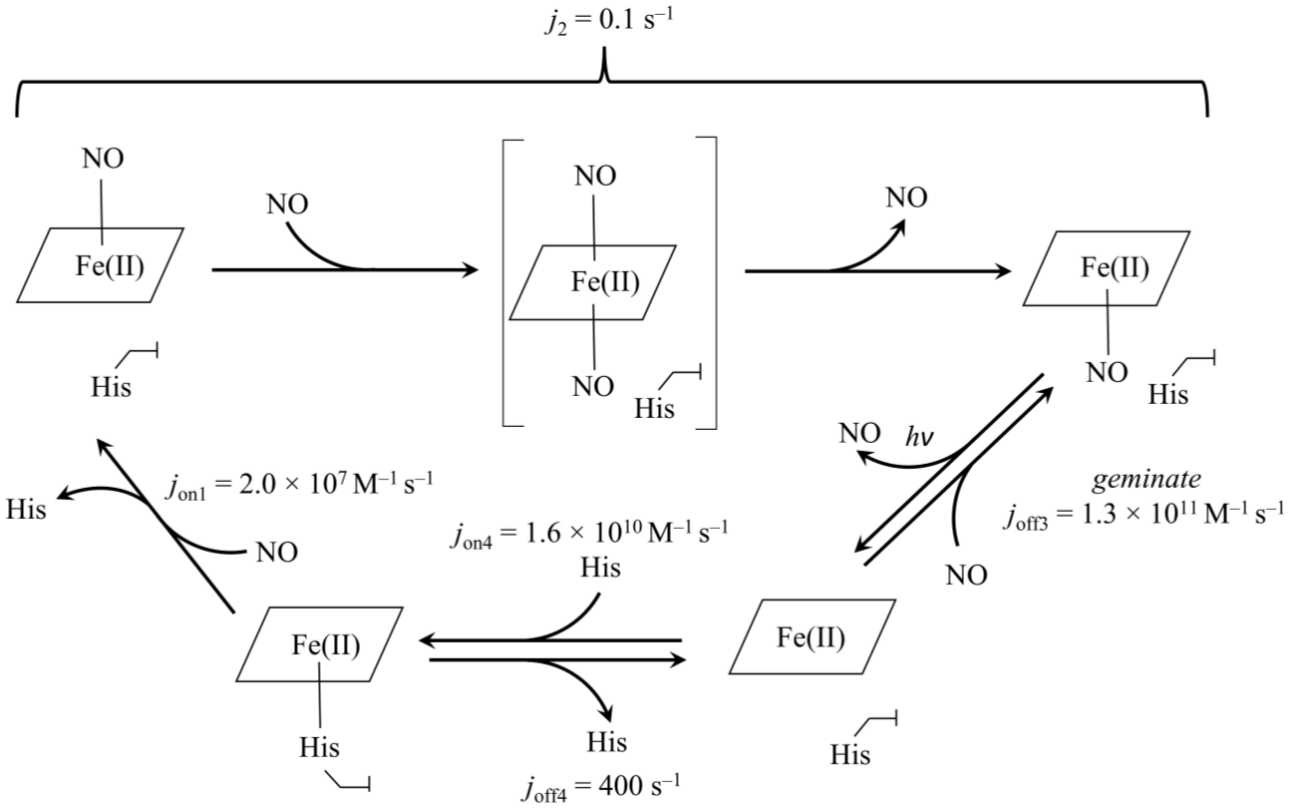
**Nitrosylation and denitrosylation reactions of ferrous CL-hhcyt *c***. For details, see the text.

NO binding to the ferrous CL-hhcyt *c* complex is very fast (*j*’ = 2.0 × 10^7^ M^−1^ s^−1^) [80], similar to that observed for Mb and Hb (see Table 1 and Scheme 1). However, the six-coordinated His18-Fe(II)-NO form is detected only in the first few milliseconds, rapidly evolving in ~20 ms to a mostly five-coordinated species, likely corresponding to the Fe(II)-NO species [80] (see Scheme 1). This indicates that the equilibrium strongly favors the five-coordinated form; the dissociation rate of His18 from the intermediate His18-Fe(II)-NO species is >50 s^−1^. Unlike Hb and Mb, additional transient species have been observed. An initial red shift to a species with a peak absorbance at λ = 420 nm, characterized by a NO-independent rate of ~7 s^−1^, was found, likely reflecting the formation of a weak six-coordinated form (i.e., X-Fe(II)-NO, where X has been hypothesized to be an internal ligand; see Scheme 1). Subsequently, the very slow formation of the final five-coordinated species with NO bound at the proximal heme side (i.e., NO-Fe(II)) has been proposed to proceed through the formation of a transient bis-nitrosylated heme [82] (Scheme 1). This behavior is different from that observed for site-specific mutants of hhcyt *c* (such as Met80Ala), which show fast NO binding but which result in a stable six-coordinated form (i.e., His-Fe(II)-NO) as the final product [145]. Therefore, (*i*) in the CL-hhcyt *c* complex, the entire tertiary structure is altered well beyond the cleavage of the Met80-Fe bond [27], and (*ii*) the CL-induced conformational changes cause a significant modification of the proximal side of the heme pocket, dramatically decreasing the energy of the His18-Fe bond, possibly facilitating NO access to the proximal side [82].

The high flexibility of hhcyt *c* within the CL-hhcyt *c* complex makes it more suitable for modulation. The NO-linked conformational changes observed at both the distal and proximal sides suggest a potential role for the nitrosylated CL-hhcyt *c* complex as a messenger for changes in hhcyt *c* action(s) when bound to CL. This implies that hhcyt *c* could serve as a NO sensor in apoptosis. In addition to its effects on mitochondrial respiration [146], NO may modulate the peroxidase activity of cyt *c* through its interaction with the heme-Fe atom, indicating that the interaction of NO with either the proximal or distal side of hhcyt *c* may represent two distinct aspects of its modulatory role. Consequently, a potential link between NO levels and apoptosis could emerge, opening a new scenario on the role of hhcyt *c* in the apoptotic process.

## 4. Concluding Remarks

This survey provides a comprehensive understanding of the diverse geometry of NO binding to heme proteins, focusing on the ligand interaction with the heme-Fe in the ferrous state and the consequent *trans* effect on the His-Fe bond. Several examples demonstrate NO-dependent alterations in the heme binding geometry, ranging from the nitrite-induced degradation of human Hb [147,148] to the AHSP-mediated stabilization of Hb α-chains [149]. Furthermore, several other heme proteins exhibit NO-dependent conformational transitions, including plant [150] and bacterial NO sensors [151], even though their mechanisms are somewhat similar to those depicted in this survey. In general terms, when NO interacts with the Fe(II) atom, it acts as an electron acceptor, favoring electron back-donation from Fe(II) to NO through a π bond, resulting in the bent geometry of the diatomic ligand [152]. Binding of NO to the distal side of the heme pocket applies strain in *trans* to the His-Fe(II) bond, leading to either the cleavage or the extreme looseness of the His-Fe(II) bond. This disrupts the link between the heme and the protein moiety. Through this mechanism, NO transmits a signal related to its environmental levels, triggering alternative activities of heme proteins, such as cGMP production in sGC [153] or lipid peroxidation in CL-hhcyt *c* [141].

The cleavage of the proximal His-Fe(II) bond may further contribute to signaling, specifically through NO binding to the proximal side of the heme. This event has been demonstrated in bacterial heme sensors, such *Ax*cyt *c*’, and postulated for sGC and CL-hhcyt *c*, providing insights into NO-based signaling. In *Ax*cyt *c*’, NO binding to the proximal pocket (strengthened by a hydrogen bond with the positively charged Arg124) forces the proximal His120 into a position that induces conformational changes in the protein, thus rendering NO binding a specific signal for a protein structural change.

Interestingly, artificial heme model compounds and heme proteins with optimal thermodynamic and kinetic parameters for ligand binding and catalysis may be designed in silico by artificial intelligence for biotechnological and therapeutic applications [154]. In this respect, the designed model of the high-affinity heme-binding protein dnHEM1 reveals excellent agreement with the 1.6 Å X-ray crystal structure. dnHEM1 displays a helical solenoid scaffold trapping the heme, an axial His residue as the fifth heme coordination ligand, a vacant heme six-coordination site representing the catalytic center, and a tunable distal pocket for substrate binding [155].

In conclusion, NO emerges as a powerful messenger due to its small size, diffusion properties across tissues (allowing access to even restricted sites), and chemical reactivity, which exerts significant stress upon binding to functional groups. These features highlight the relevance of NO interaction in various pathologies, particularly those associated with blood flow regulation and oxygen supply to poorly oxygenated tissues, such as the retina. This opens the way to focus on the regulation of NO levels in these tissues and to their connection with sGC activity, which appears very promising for a therapeutic approach in the case of glaucoma [19,24,156].
